# Healing the next generation: an adaptive agent model for the effects of parental narcissism

**DOI:** 10.1186/s40708-020-00115-z

**Published:** 2021-03-02

**Authors:** Fakhra Jabeen, Charlotte Gerritsen, Jan Treur

**Affiliations:** Social AI Group, Vrije Universiteit Amsterdam, Amsterdam, The Netherlands

**Keywords:** Narcissism, Parental influence, Reified architecture, Social contagion, Adaptive agent

## Abstract

Parents play an important role in the mental development of a child. In our previous work, we addressed how a narcissistic parent influences a child (online/offline) when (s)he is happy and admires the child. Now, we address the influence of a parent who is not so much pleased, and may curse the child for being the reason for his or her unhappiness. An abusive relationship with a parent can also cause trauma and poor mental health of the child. We also address how certain coping behaviors can help the child cope with such a situation. Therefore, the aim of the study is threefold. We present an adaptive agent model of a child, while incorporating the concept of mirroring through social contagion, the avoidance behaviors from a child, and the effects of regulation strategies to cope with stressful situations.

## Introduction

“…phenomena are best understood when placed within their series, studied in their germ and in their over-ripe decay.” [1]

The behavior of a parent can contribute significantly to the development of a child, from childhood to adulthood, as a parent is perceived as a role-model by a child [2]. Inspired by their parents, they often try to mimic them in everything they do. This learning process plays an important role in the development of their personality, and when it comes to the parental narcissism, startling effects can be expected [2, 3]. Hence, it would be interesting to see whether parent behavior would make a child to put effort to mirror the parent’s behavior or, oppositely, not to behave like the parent [4].

In the literature, it is indicated that the self-esteem of a child is positively correlated with the feedback received from the approval or disapproval of a parent [5]. Narcissistic parents project their inflated self-views onto their children, who internalize these experiences in an unconscious manner, resulting in a mimicking behavior [4]. When children are overvalued or indicated to have superiority over others, they tend to develop narcissism [6]. Moreover, when it comes to the self-protection of a highly narcissistic parent, (s)he leaves his/her children abandoned and often treat them with aggression/abuse [7]. The victims of unhappy behaviors or narcissistic abuse can only survive if they are able to cope with such situations [8, 9].

Causal modeling is a field of artificial intelligence which can address real-world processes related to different domains such as psychology, sociology, cognitive or neuroscience; e.g., [10, 11]. Causal models for real-world processes are designed to address the triggering of certain events leading to a certain behavior or a process with certain effects. For example, how a smile on a single face can make others smile. In our earlier work, we presented an adaptive agent model of a child, who is influenced by a narcissistic parent. We tried to explain that when sensing that the parent is happy, a child may internalize and may mimic this parent. We also discussed how social media can play a role in the reflection of such behavior [12]. However, narcissistic parenting has a dark side as well, which needs to be addressed. By this we mean, how an unhappy (or angry) narcissistic parent can influence a child. It is also interesting to see, how coping mechanisms can help a child to survive.

More specifically, in this paper, in addition to the agent model of a child who is influenced by happy parenting (offline/online), we address (a) how unpleasant parenting can influence child behaviors, and (b) how coping can help the child. Thus, the paper is organized in five sections. In Sect. 2, we present the state-of-the-art literature related to the behaviors of a child when influenced by a parent and how coping behaviors and strategies can help in stressful situations. Section 3 presents methods and methodologies applied along with the adaptive model of a child. Section 4 discusses the simulation experiments, while Sect. 5 discusses and concludes the paper.

## State-of-the-art literature

Much research literature is available regarding the behavior of children, and addresses the social and mental development of a child under the influence of a parent [3, 6, 13]. This section presents the related work in three streams. Firstly, it discusses the psychological and social effects of a narcissistic parent on a child. Secondly, it presents how a parent can influence a child’s neurology. Lastly, it discusses coping mechanisms for parental narcissism along with AI-based approaches available till now.

### Psychological and social effects of parental narcissism

Narcissism is characterized by the mythological figure ‘Narcissus’, who fell in love with his own reflection. There is a clear distinction between self-esteem and narcissism. The former is related to the sense of self-worth of a person, while the latter is related to the acute concern for self-admiration [14]. Parent–child attachment plays an important role in the development of the psychology and nature of a child [15]. Parental warmth and affection produce compassion, and empathy in behaviors [15], which results in an adult with a high self-esteem. In [6] it is described how a study was conducted with four waves with the gap of 6 months, to study how parental overvaluation influences a child. The results indicate that overvaluation can induce narcissism in a child [6]. The self-inflation hypothesis states that the over-admiration of a child makes him perceive, how others look at them. The processes of having a sense of superiority lead to narcissism [16].

Moreover, the concept of using social media or web playgrounds is not very uncommon in children, and is thought to be an interesting forum [17]. Such forums allow them to interact socially, play together, form relationships and share some exciting stuff [18] with other peers. Mimicking behavior can be reflected while using social media, this is because of internalizing experiences which they have formed from the mutual parent–child interaction [2]. Internalization experiences cause them to form an image about themselves, which is in a way a projection of behaviors from their caregivers (e.g., parents). Another study indicates that children mimic the grandiosity of their mothers [4], however, this may reduce with age or maturity [19].

While discussing the darker side of narcissistic parenting, a child can be affected badly during childhood [20]. It leads to low-esteem or a diminished sense of self [21]. They experience trauma and may go towards anxiety and isolation [8] or even may think of suicide [9]. Constant anxiety and stressful situations can lead a person to mental sickness and psychological alterations [22]. However, researchers also proposed a few strategies to live a healthy life which is only possible with the passage of time, or when a child is able to learn and to apply them [9].

### Cognitive effects of parental narcissism

From a cognitive and neurological perspective, parental influence is noticed from the early years of a child [23]. Parent–child interaction has been witnessed to produce variations in brain volume and gray matter [23, 24]. Also, another study indicates that there is more perceptual similarity of actions between parents and their children for certain situations [25]. This results in the involvement of similar brain regions, for example, within the prefrontal cortex (PFC), the temporal lobes, and the insula, when evaluating any comment or compliment [26]. Overvaluation is considered by a child as a compliment, thus it induces reward-seeking behavior, as is shown by activations in the anterior cingulate cortex (ACC) and ventral striatum [26–28]. Moreover, when a child uses social media, sharing certain content takes place. Also here, the target may be to get admiration or acknowledgment, so that hormones like dopamine are released [27].

On the downside of the parental narcissism, a child can feel shivers due to stress and anxiety [9]. Here, γ-aminobutyric acid (GABA) receptors get activated when a person is experiencing anxiety [29], while higher levels of cortisol are observed in situations of stress [30]. Some studies also report the feeling of guilt or depression with the lack of satisfaction, thus leading to anxiety; e.g., [9].

### Coping with parental narcissism

Children of narcissistic parents often suffer from life-lasting behavioral issues, and may have a high vulnerability [31, 32]. They may have experienced traumatic situations, especially when they were unable to please their parent [8]. As a parent–child relationship is considered as a lifelong relationship, therapies or regulation strategies should be used to endure unpleasant parent–child experiences. A child may seek help from others, who can be therapists or, can learn to survive by him or herself through different problem-solving techniques. For example, a few techniques from Cognitive Behavioral Therapy (CBT) can effectively reduce depression and stress [33]. This therapy is traditionally done by therapists, however, there are individuals who can learn it and can have the best use of it at their own [34]. Examples are having a journal or harmonizing themselves rather than becoming a scapegoat [34].

Some Artificial Intelligence-based approaches are available to detect narcissism through text [35, 36], without studying causal relationships for behaviors. Temporal–causal modeling is a field which is widely used to address causal relationships for behaviors, for example, in biological, social or cognitive domains. It can model how one person influences another through the concept of social contagion. Also, it discloses how over time the environmental factors can influence the moods, brain and psychology of a person [11, 37]. Previously, a temporal–causal model was designed for a narcissist, which reflected how the brain of a narcissist may work, and the reaction (s)he may show over some feedback [38]. Moreover, in our previous work, we presented an agent model of a child, who shows a high tendency to become a narcissist like a parent [12] or may also learn to act otherwise. Here, we extend our work [12] by studying the influence of a narcissistic parent, particularly when this parent is not happy. Also, we address how problem-solving skills or self-therapies can help the child to survive.

## Methods and methodologies and the adaptive agent model

Causal modeling is a well-known approach in the field of Artificial Intelligence, which is widely used to represent the causal factors underlying behaviors in the real world. These models have variables as their basic elements, with certain causality relations between them that together form a causal network. Each variable represents an occurrence of an event (e.g., “he won presidential elections” or a mental state), which leads to a change in a certain scenario (e.g., “he became a president”) [39]. *Temporal–causal network modeling* distinguishes itself from static causal modeling by adding a temporal perspective to it. Moreover, *adaptive* temporal–causal network models address the changes in the strength of causal connections or other network characteristics over time. They are widely used to address a variety of neural, mental, biological, social network models in many domains [11]. This section describes the adaptive network modeling approach using a so-called reified or self-modeling network architecture, which was used to design our adaptive model.

A *reified or self-modeling network architecture* is a multilevel architecture, in which a temporal–causal network is presented at the base level, and the adaptiveness of the model is presented in the form of self-models at higher (reification) levels of the architecture. The temporal–causal model at the ***base level*** contains ‘states’ as vertices connected with a set of ‘connections’ as edges between them. Here states *Y* have *activation levels Y(t)* that vary over time *t*. To illustrate it further, consider a connection *X* → *Y*, representing state *X* influencing state *Y*. The activation level of *Y* is the result of an *aggregated causal impact* via all incoming connections from states *X*_*i*_. This aggregated causal impact is computed by a *combination function*, applied to the single causal impacts.

$${\mathbf{impact}}_{{X_{i} ,Y}} (t) = {\varvec{\upomega}}_{{X_{i} ,Y}} X_{i} (t)$$

defined by the *connection strengths* **ω**_*Xi*,*Y*_ of the incoming states *X*_*i*_ and their *state values X*_*i*_*(t)* at a certain time *t*. Therefore, for each state *Y* in a temporal–causal network model, the following *network characteristics* are specified:

- **Connectivity characteristics: connection weights ω**_*X,Y*_

This represents how strong a state *X* can influence state *Y*. The magnitude varies between 0 and 1, however suppression from a state is represented by a negative connection weight.

- **Timing characteristics: speed factors η**_*Y*_

This represents how fast state *Y* can get influenced by the aggregated causal impact from the incoming connections. The range is normally between 0 and 1, showing low and high, respectively. In this way, there is no need to assume (as in other cases often is silently done) that all states behave synchronously with the same speed.

- **Aggregation characteristics: combination functions c**_*Y*_**(..)**

This is used to compute the aggregated causal impact of all of the incoming connections. To accomplish this, a predefined function (e.g., identity, alogistic, scaled sum, and so on) from a Combination Function Library can be used, where a custom function can also be defined and added to this library.

The network characteristics for all states (introduced above) define a full specification for a temporal–causal network model which can be used as input for an available dedicated software environment. The causal impact on state *Y* at time *t* determines the value of *Y* at time *t* + Δ*t*; this is computed by:

$$Y\left(t+\Delta t\right)=Y\left(t\right)+{{\varvec{\upeta}}}_{{\varvec{Y}}}\left[\mathbf{a}\mathbf{g}\mathbf{g}\mathbf{i}\mathbf{m}\mathbf{p}\mathbf{a}\mathbf{c}{\mathbf{t}}_{{\varvec{Y}}}\left(t\right)-Y\left(t\right)\right]\Delta t$$

and

1 $$\frac{\mathbf{d}Y\left(t\right)}{{\varvec{d}}t}= {{\varvec{\upeta}}}_{{\varvec{Y}}}\left[\mathbf{a}\mathbf{g}\mathbf{g}\mathbf{i}\mathbf{m}\mathbf{p}\mathbf{a}\mathbf{c}{\mathbf{t}}_{{\varvec{Y}}}\left(t\right)-Y\left(t\right)\right],$$

where **aggimpact**_*Y*_(*t*) is computed as:

$${\mathbf{a}\mathbf{g}\mathbf{g}\mathbf{i}\mathbf{m}\mathbf{p}\mathbf{a}\mathbf{c}\mathbf{t}}_{Y}\left(t\right)= {\mathbf{c}}_{Y}\left({\mathbf{i}\mathbf{m}\mathbf{p}\mathbf{a}\mathbf{c}\mathbf{t}}_{{X}_{1},Y}\left({\varvec{t}}\right), ..., {\mathbf{i}\mathbf{m}\mathbf{p}\mathbf{a}\mathbf{c}\mathbf{t}}_{{X}_{k},Y}\left({\varvec{t}}\right)\right) ={\mathbf{c}}_{Y}\left({{\varvec{\upomega}}}_{{X}_{1},Y} {X}_{1}\left({\varvec{t}}\right), ..., {{\varvec{\upomega}}}_{{\mathrm{X}}_{k},\mathrm{Y}}{\mathrm{X}}_{k}\left({\varvec{t}}\right)\right).$$

*Adaptivity of a network model* comes in when these characteristics change over time. This is represented by self-models at higher levels, for example representing ***first-order adaption principles*** (modeled as Level II) and ***second-order adaption principles*** (modeled as Level III). In other words, an ***n***th-order adaptive network model is modeled as an ***n*** + 1 leveled network, and can also be represented mathematically (Appendix). For example, an adaptive connection weight **ω**_*X,Y*_ is modeled by adding a self-model state **W**_*X,Y*_ that represents the (adaptive) value of **ω**_*X,Y*_. Such states, called *reification states* or *self-model* states, are the basis of self-models within a network that represents part of the network’s own network structure by some of its states. Here, we use a second-order adaptive reified network architecture to address the adaptive agent of a child who is interacting with and influenced by a parent.

### Level I: the base network level

**Fig. 1:**
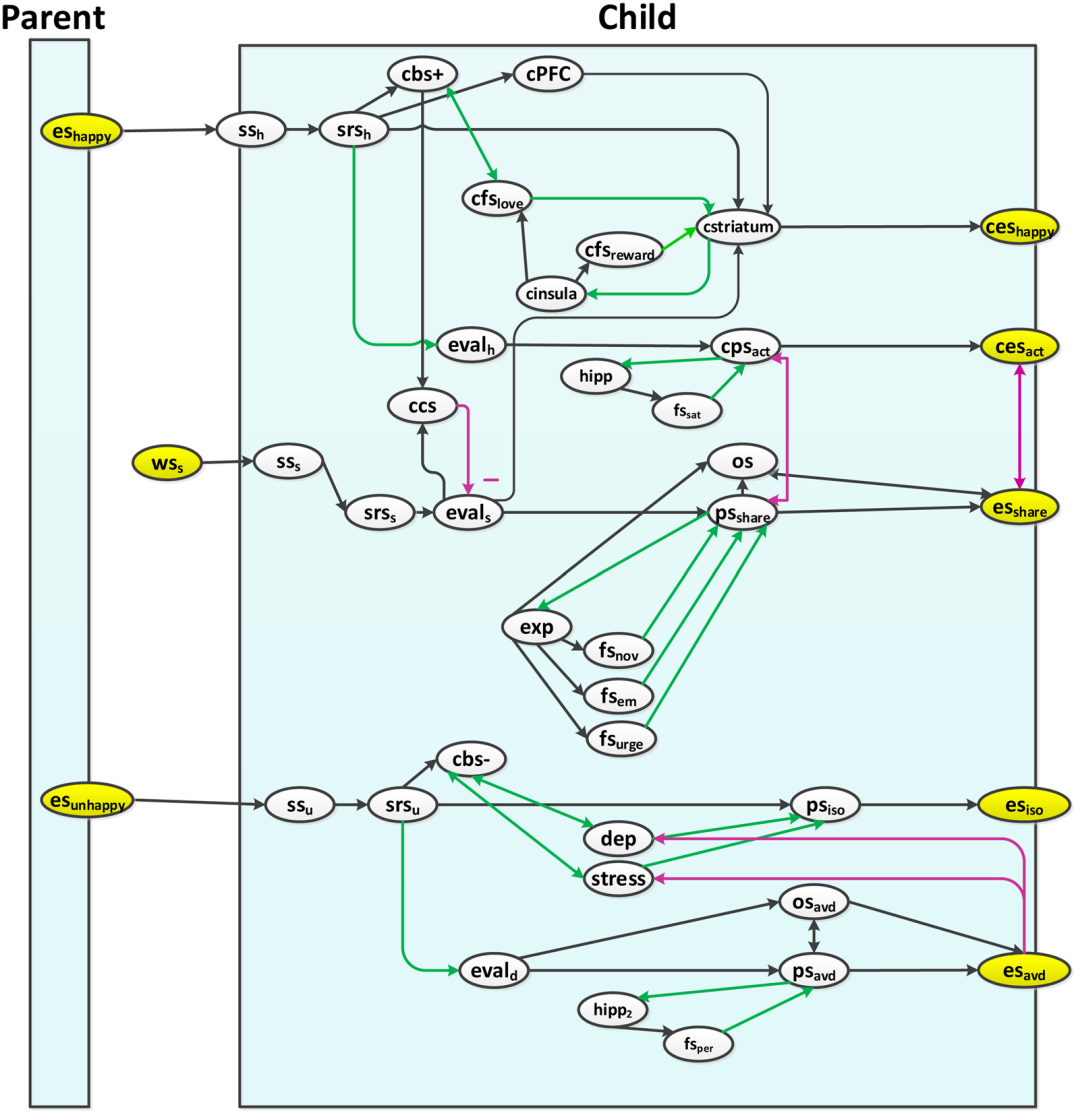
Connectivity of the base level (Level I) of the network model of a child influenced by parent behavior

This section presents the base network model (Level I), which depicts the mental organization of a child under the influence of a parent. The base network is designed according to the literature discussed in Sect. 2 and has 39 states, in Fig. 1 shown as the base plane of the model. A categorical explanation of each of the states is presented in Table 1. A state can have three types of incoming connections:

- Black arrows indicating a positive connection, with possible connection weight values between [0,1].
- Purple arrows indicating a negative connection with weight values between [-1,0].
- Green arrows show the adaptive connections which lead to adaptive behavior and will be explained further in Sect. 3.2.

**Table 1:** Categorical explanation of states of the base model (Level I)

Categories		References
**Stimulus states and sensing:**		
es_happy_ es_unhappy_ ws_s_ ss_s_ srs_s_	Narcissistic parent shows happy Narcissistic parent shows unhappy Using social media Sensor state for the child for s Representation state of the child for s	“the representation of the world external to the body can come into the brain only via the body itself” [40]
**Social contagion related states of the child:**		
cbs + cstriatum cPFC csfs_love_ cfs_reward_ ces_happy_	Belief state of the child Striatum: Brain part of the child Prefrontal Cortex: Brain part of the child Feeling of self-love (Amygdala) Feeling of self-reward (Amygdala) Execution state expressing happiness for the child	“ yet familiarity.. infants copy more actions of a familiar, compared to an unfamiliar model” [2] “mothers show high self– child overlap in perceptual similarity in the FFA regardless of their relationship quality with their child” [25]
**Non-narcissistic behavior related states:**		
eval_h_ cps_act_ ces_act_ hipp_1_ fs_sat_	Evaluation state for analyzing behaviors Preparation state Execution state Hippocampus: Brain part for memories Feeling of satisfaction	“adolescents was associated with neural activation in social brain regions required to put oneself in another’s shoes” [25]
**Social Media related states:**		
eval_s_ os ps_share_ es_share_ exp fs_i_	Evaluation of the input, based on belief Ownership state Preparation state Execution state Experience Feeling states: *i* = novelty(nov)/emotion (em) /urge	“Emotion then facilitates behavior that is in line with our concerns” [41]
**Unhappy reaction related states:**		
ss_u_ srs_u_ cbs- fs_*i*_ eval_d_ os_avd_ ps_*i*_ es_*i*_ hipp_2_	Sensor state for sensing unhappiness Representation state of sensed unhappiness Low belief about himself (Low esteem) Feeling states: *i* = depression(dep)/stress (str)/persuasive (per) Evaluation of parent as a narcissist Ownership state of avoidance behavior Preparation states: *i* = isolation (iso)/avoiding parent (avd) Execution states: *i* = isolation (iso)/avoiding parent (avd) Hippocampus: Brain part for memories	“Emotion then facilitates behavior that is in line with our concerns” [41] “children who perceived their fathers to be highly critical.. engage in insulting name calling, and to use guilt arousal and love withdrawal … unstable SE indicated that their fathers less frequently talked about the good things” [42] “the possibility of inherited narcissism and employing narcissistic parenting strategies… analyze themselves constantly and protect their emotional motherhood constantly” [9]

According to the literature addressed in Sect. 2, a child can sense three kinds of inputs from his or her surroundings:

- Happiness of a narcissistic parent.
- An angry or unpleasant behavior from a parent.
- Reactions on social media.

In Fig. 1, only two states of a narcissistic parent are shown, which act as an input to our model of a child, i.e., es_happy_ (when the parent is happy) and es_unhappy_ (when the parent is angry or not happy). For details of narcissistic parent behaviors, we refer to [38].

Firstly, a narcissistic parent being happy, may stimulate the child to mimic the parent’s behavior. This stimulus es_happy_ is observed by a child, which activates the sensing states ss_h_ and the sensory representation states srs_h_. An example can be.

“The parent looks happy when noticed in the crowd, also a child feels good when noticed.”

In such a case, the child believes positive about himself or herself (by cbs +); different brain parts within the PFC and the insula are activated along with the amygdala. This leads to the activation of feelings related to self-reward and self-love, and make this behavior aware. As a result, like the parent, the child exhibits narcissism: ces_happy_. This mimicking behavior is a form of social contagion.

Also, it is quite possible that a child tends not to agree and align with the parent’s behavior, but rather acts differently. An example can be.

“A narcissistic parent looks happy when noticed in the crowd, however the child may prefer to remain unnoticed.”

Understanding the narcissistic behavior and nature of a parent comes with maturity or age and experience, modeled as hipp, and as the child conceptualizes it, modeled as eval_h_. Therefore the child may prefer not to mimic the parent, but rather chooses his/her own behavior which pleases his or her mind. This also reflects that narcissism may fade away as the child grows older and counts him or herself responsible for his/her own actions, modeled by ownership state os.

Secondly, a displeased narcissistic parent may influence a child in a different way. An example can be.

“an angry narcissistic parent had some conversations with his or her child which may lead the child towards isolation or an escape.”

This unpleasant stimulus es_unhappy_ is sensed, via ss_u_ and srs_u_, by the child which lowers his or her esteem-belief: cbs-. So, thinking him or herself as the source of displeasure to the parent, the child feels rejected and upset, causing him or her depression and stress feeling fs_dep_ and fs_stress_. As a result, this may lead the child towards isolation: ps_iso_ and es_iso_.

Another possibility is the realization of a parent’s ‘displeasing behavior as a disease or abnormality’, modeled by eval_d_, which may save the child from hurting him or herself further. This is not a one day process, rather it is learned over time through the experience and age, due to the parent–child relationship memories hipp_2_ and the feelings of persuasiveness fs_per_. So, the child is consciously (modeled by os_avd_) struggling to avoid via ps_avd_ and es_avd_ or trying to have some therapy to regulate him or herself.

Thirdly, when using social media, a child may tend to share some content with the influence of a narcissistic parent under two possibilities. Either the child shares for his or her narcissistic pleasure or if the child is not mirroring the parent then (s)he shares his or her own expression. When encountering a post (through ws_s_, ss_s_, and srs_s_), the child can evaluate (by eval_s_) its contents ‘to be interesting’. The sharing tendency of this content is based upon three attributes represented by their respective feelings: novelty fs_nov_; emotional value fs_em_ attached to the content; and the urge fs_urge_ to share the content. These three attributes are learnt over the passage of time, and as a result, the child shares the content (via ps_share_ and es_share_) over the social media. This sharing behavior may or may not influence the self-rewarding behavior or states of the child. It is to be noted here that initially the child’s control state ccs controls the sharing phenomena based upon beliefs influenced by the parent. However, later the involvement of the ownership state os indicates that this action is self-attributed.

### Level II: first-order adaption level

**Fig. 2:**
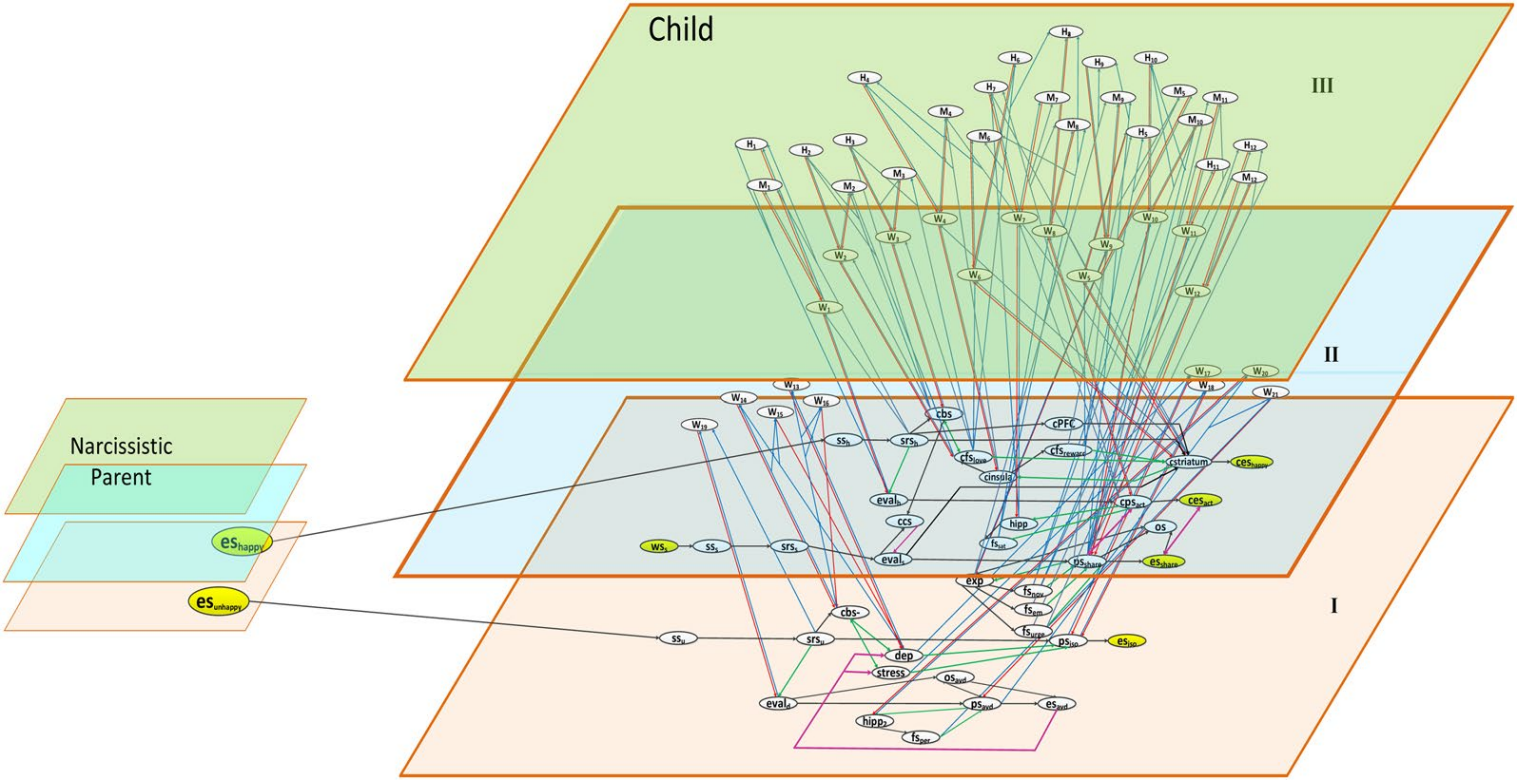
Second-order reified network model of a child under the influence of a parent

In this section, we will discuss the first-order adaption level represented at Level II. It is related to the ability to learn certain behaviors over a period of time (e.g., age/maturity), known as *plasticity* or *Hebbian learning* [43]. In this case, the connection strengths tend to change over time. Therefore, they are represented by **W**-states **W**_*i*_ (where *i* = 1 to 21) at Level II that form a (first-order) connectivity self-model within the network. These W-states determine the connection strength of the connections at Level I. The particular connections that are addressed, are depicted as green arrows at Level I. As most states, these **W**-states change over time so that the base-level connections indeed get weights that adapt over time; this is specified according to the Hebbian learning adaptation principle, expressed by combination function **hebb**_**μ**_**(..)** defined at the end of Sect. 3. Some of the network characteristics of this first-order self-model based on the **W**-states are adaptive as well, in particular, the timing characteristics (the learning rates for the Hebbian learning) and the aggregation characteristics (for the persistency **μ** of the learned effects) of the Hebbian learning modeled by this self-model; this will be discussed later in Sect. 3.3. The connectivity of the complete model with its leveled architecture is shown in Fig. 2.

To explain this behavior, consider W_10_ (or $\rm W_{{\rm fs}_{\rm nov},{\rm ps} _{\rm share}}$). Here, two states fs_nov_ and ps_share_ are connected through fs_nov_  ps_share_, which act as presynaptic and postsynaptic states, respectively. The learning behavior is observed when sending state fs_nov_ transmits a signal to the receiving state ps_share_ making it either more or less likely to fire its own action potential (against a certain threshold). These action potentials are not fired promptly; instead, they can last for a while before they can dissipate. Due to this behavior, the strength of the connection fs_nov_  ps_share_ can get stronger or weaker and can be learned over time in that way. Here, the input from the connected states to the corresponding **W**-state is shown by upward blue arrows (from Level I to Level II), while the influence of a **W**-state is shown by downward red arrows (from Level II to Level I), forming a circular causation. Table 2, enlist the **W**-states, along with their related connections. For more details related to the modeling such a behavior see [11, 43].

**Table 2:** Explanation of states in level II and III

States per level			References
**Level II (Plasticity/Hebbian learning for Omega states):**			
W_1_: W_2_: W_3_: W_4_: W_5_: W_6_: W_7_: W_8_: W_9_: W_10_: W_11_: W_12_: W_13_: W_14_: W_15_: W_16_: W_17_: W_18_: W_19_: W_20_: W_21_:	${\rm W}_{{\rm srs}_{\rm h},{\rm eval} _{\rm h}}$ ${\rm W}_{{\rm bs},{\rm fs} _{\rm love}}$ ${\rm W}_{{\rm fs}_{\rm love},{\rm bs} }$ W_striatum,insula_ ${\rm W}_{{\rm fs}_{\rm reward},{\rm striatum} }$ ${\rm W}_{{\rm fs}_{\rm love},{\rm striatum} }$ ${\rm W}_{{\rm ps}_{\rm sat},{\rm hipp} }$ ${\rm W}_{{\rm fs}_{\rm sat},{\rm ps} _{\rm act}}$ ${\rm W}_{{\rm ps}_{\rm share},{\rm expo}}$ ${\rm W}_{{\rm fs}_{\rm nov},{\rm ps} _{\rm share}}$ ${\rm W}_{{\rm fs}_{\rm em},{\rm ps} _{\rm share}}$ ${\rm W}_{{\rm urge},{\rm ps} _{\rm share}}$ W_cbs,dep_ W_dep,cbs_ W _cbs,stress_ W_stress,cbs_ ${\rm W}_{{\rm dep},{\rm ps} _{\rm iso}}$ ${\rm W}_{{\rm stress},{\rm ps} _{\rm iso}}$ ${\rm W}_{{\rm srs}_{\rm u},{\rm eval} _{\rm d}}$ ${\rm W}_{{\rm ps}_{\rm avd},{\rm hipp} _{\rm 2}}$ ${\rm W}_{{\rm fs}_{\rm per},{\rm ps} _{\rm avd}}$	for srs_h_ eval_h_ for cbs + cfs_love_ for cfs_love_ cbs + for cstraitum cinsula for cfs_reward_ striatum for cfs_love_ striatum for ps_sat_ hipp for fs_sat_ cps_act_ for ps_share_ exp for fs_nov_ ps_share_ for fs_em_ ps_share_ for urge ps_share_ for cbs dep for dep cbs for cbs stress for stress cbs for dep ps_iso_ for stress ps_iso_ for srs_u_ eval_d_ for ps_avd_ hipp_2_ for fs_per_ ps_avd_	2–6: “Potentiation in the striatum depends not only on strong pre- and postsynaptic activation … reward prediction … modify behavior” [27] 1; 7–8: “Older people,… might be less likely to harbor narcissistic traits. This again suggests that narcissism should decrease with age” [19] 9–12: “Emotion then facilitates behavior that is in line with our concerns” [41] 14–21:“From speech, perception, and interpersonal interactions with his primary caregivers, he had to protect himself cognitive processes, both conscious and non-conscious” [8]
**Level III (Meta-Plasticity/Learning rate and persistence):**			
M_*i*_: H_*i*_:	Persistence Learning rate	for *i* = W_*j*_: *j* = 1,…,12 for *i* = W_*j*_: *j* = 1,…,12	“Metaplasticity refers to neural changes that are induced by activity at one point in time and that persist and affect subsequently induced LTP or LTD” [44]

### Level III: second-order adaption level

This level III represents the adaptation of some of the network structure characteristics describing the first-order self-model at the first-order adaption level, i.e., Level II. This makes that not only the **W**-states at Level II change over time, but also the mechanism through which the **W**-states change is changing. This (Hebbian learning) mechanism may learn/adapt over time, which represents *plasticity of plasticity* or *metaplasticity* ([45], Schmidt et al. [46]). This metaplasticity is applied in particular to the learning rate (used at level II as a timing characteristic of the self-model) and persistence factor (used at level II as an aggregation characteristic of the self-model) of the Hebbian learning mechanism. More specifically, it is achieved by explicitly modeling at Level III adaptive persistence factors **μ** and adaptive learning rates **η** by second-order reification states **M**_*j*_ and **H**_*j*_ (where *j* = 1 to 12) that in this way constitute a second-order aggregation and timing self-model for the first-order connectivity self-model at Level II. These states specify how the mechanism of synaptic transmission can be influenced or controlled through hormonal release and neurotransmission [11, 45]. This second-order adaptive behavior is modeled through upward and downward inter-level connections, having the same pattern as we discussed for Level I and II (Sect. 3.2). By this we mean, that these states of second-order reification or self-model, take input from their respective presynaptic and postsynaptic states from Level I, and influence the involved W-states at Level II.

To illustrate it further, consider the self-model states **M**_10_ and **H**_10_ which play an adaptive role in the dynamics of **W**_10_ (${\rm W}_{{\rm fs}_{\rm nov},{\rm ps} _{\rm share}}$). They receive a causal input from the respective presynaptic (fs_nov_) and postsynaptic (ps_share_) states along with **W**_10_, which is represented by the upward blue arrows. As a consequence (or circular causation), states **M**_10_ and **H**_10_ determine the persistence and speed factor of the state **W**_10_ at Level II, represented by the red downward arrows. A lower value for **H**_10_ will cause a lower speed for learning of the state **W**_10_, while a lower value of **M**_10_ will be responsible to lower persistence of **W**_10_ and vice versa. This will in turn control the dynamics of the connection weights of the connection fs_nov_  ps_share_; see also [11], Ch. 4, p. 110.

The network model is simulated using the reified network engine within the dedicated software environment developed in MATLAB, by providing a declarative specification (specifying declarative mathematical relations and functions) for the designed agent. This specification is in the form of role matrices, which are given as input to the engine. Each role matrix represents the network characteristics according to the role played with respect to a certain state. For example, for the *connectivity characteristics* base matrix (**mb**) contains for each state *Y* information about the incoming connections to *Y*. Similarly, connection weight matrices (**mcwv** and **mcwa**) indicate the connection weights of all of the states in the model. They can be adaptive by nature (specified by **mcwa**) or can be constant (specified by **mcwv**). For the *timing characteristics*, speed factor matrices **msa** and **msv** represent the adaptive and non-adaptive speed factors, respectively. Finally, for the *aggregation characteristics*, combination function weight matrices (**mcfwa** and **mcfwv**) and combination function parameter matrices (**mcfpa** and **mcfpv**) specify the combination functions along with their weights and the related parameters, respectively, both for the adaptive and non-adaptive ones.

To illustrate the declarative specifications of the model further, let us consider state ps_share_, which has 4 incoming connections. One of them is the non-adaptive connection eval_s_** → **ps_share_, while the rest connections are adaptive by nature: fs_nov_  ps_share_, fs_em_  ps_share_, and fs_urge_  ps_share_. So, in **mb** we specify the names (by their index) of all of four states that have causal influence on state ps_share_. As we have one out of four non-adaptive connections, role matrix **mcwv** will only have a connection weight for one incoming connection, i.e., for eval_s_** → **ps_share_ (weight value = 0.5). The rest of the adaptive connections are specified by the names (indicated by their indices) of the respective **W**-states in **mcwa**, indicating that the respective **W**-states are responsible for the change of the connection strength over time (See Fig. 2). The speed factor for ps_share_ is specified in **msv**, and has the value 0.6. The activation level for ps_share_ is computed through the alogistic combination function which is specified through **mcfw**. Finally, to set the parameters for the alogistic combination function **mcfpv** is used, containing the parameter values for the alogistic function defined as:

$${\mathbf{alogistic}}_{\sigma ,\tau } (V_{1} , \ldots ,V_{k} ) = [(1/(1 + {\text{e}}^{{ - \sigma (V_{1} + ... + V_{k} - \tau )}} )) - 1/(1 + {\text{e}}^{{\sigma{\tau } }} )](1 + {\text{e}}^{{ - \sigma{\tau } }} ),$$

where:

*V*_1_,…*V*_*k*_ represent the single impacts from the four incoming states, i.e., from eval_s_, fs_nov_, fs_urge_, fs_em_.from eval_s_, fs_nov_, fs_urge_, fs_em_.*σ* = the steepness (value = 10 for ps_share_), and *τ* = the threshold for the activation (value = 0.2 for ps_share_).

In total, the designed network model has 84 states: 39 states at Level I, while Level II and Level III have 21 **W**-states and 24 **M**- and **H**-states, respectively. To compute the activations over time, we used three type of combination functions for our network model i.e.:

- 10 states (ss_h_, srs_h_, ces_happy_, fs_sat_, ss_s_, srs_s_, ss_u_, srs_u_, fs_per_, es_avd_) use the Euclidian function, which is specified by:
  **eucl**_n,λ_ (*V*_1_*,…,V*_*k*_**)** = $$\sqrt[{\varvec{n}}]{{(V}_{1}^{n}+\dots + {V}_{k}^{{n}})/{\varvec{\lambda}}}$$,
  where
  *n* = order of the Euclidian function, and
  λ = scaling factor for normalization.
- All W-states (**W**_*i;*_* i* = 1–21) use Hebbian learning principle defined by:
  $${\mathbf{hebb}}_{{{\varvec{\upmu}}}} (V_{{1}} ,V_{{2}} ,W)\, = \,V_{1} V_{2} (1 - W)\, + \,\mu W,$$
  where
  **µ** = persistence factor for the learning,
  *V*_1_ and *V*_2_ are the activation levels of the connected states, and
  *W* = their connection weight ${\varvec{\upomega}}_{{\rm V}_{1},{\rm V}_{2}}$*.*
- Lastly, the remaining 53 states (including for the 24 states of **M** and **H** at Level III) use the alogistic function where steepness and threshold ≤ 1, also addressed earlier.

To get further insights into the declarative role matrix specification format, see [11]. Also, for the full specification of the adaptive network agent model of the child see [12].

## Simulation experiments

Setting up the simulation experiments enables researchers to study and analyze the dynamics of any real-world scenario they want to consider. In this section, in particular, we will study the dynamics of our agent model of a child under the parental influence. First, we will address how a child may behave when he senses the happiness or unhappiness of a narcissistic parent. Second, it will be addressed how a child behaves when he is using social media under the parental influence. Third and last, we address how a child can learn to cope with a narcissistic parent. One thing to be noted is that we will not discuss the behavioral changes of a parent as they are already addressed in detail in [38]. Therefore, this section is divided into three subsections with respect to different behaviors of a child:

- A child has a strong tendency to exhibit similar behaviors like the parent,
- A child shows resistance to imitate the parent behavior, and
- A child trying to cope with an unhappy narcissistic parent.

### Exhibition of narcissistic behaviors

Here, we present the behaviors of a child who is influenced by a parent. We will discuss how a happy parent nurtures narcissism in the child from childhood to adulthood years. We will discuss two scenarios: (a) parental influence without the digital world and (b) with the digital world.

#### Parental Influence ‘off’ the digital world

Most of us are aware of the idiomatic expression ‘*like mother, like daughter’*. Consider a child whose parent is a TV actor or a renowned one, always seeking appraisal and love from the society, in such a situation the child would also like to mimic that parent.

**Fig. 3:**
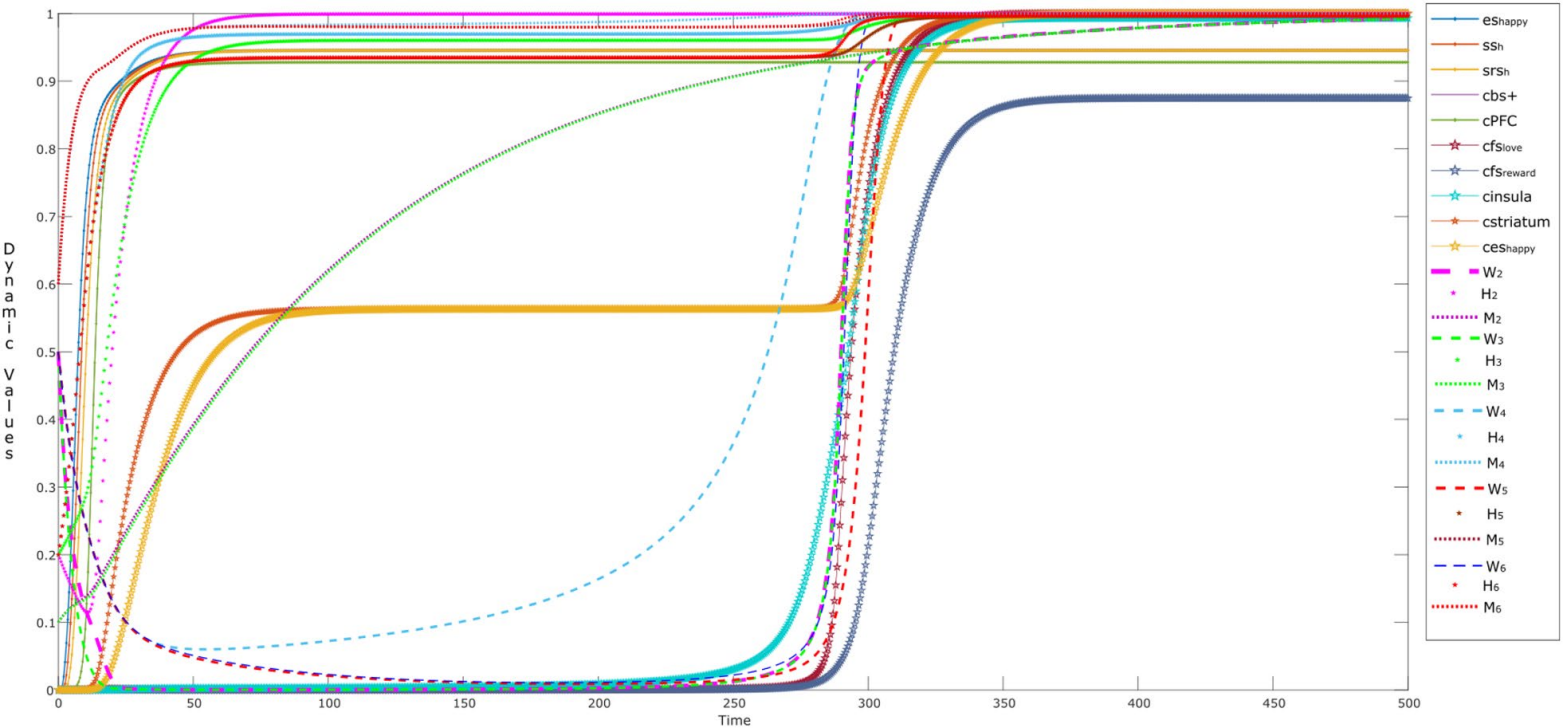
Child is mimicking his/her parent’s behavior as (s)he senses happiness (input: es_happy_), along with learning

Figure 3 shows the simulation of such an interaction between the parent and child over time. The child senses when the parent is the center of attention and looks happy (state es_happy_ tends to get a high value of 0.95 at time point *t* = 25), which is sensed through the child’s sensor state ss_h_ deep orange) and representation state srs_h_ (mustard). The child’s main source for learning human behaviors is in early years of age, and the main source to learn is from the parent or caretaker (so considering ws_*s*_ = 0). The fostering behavior of the parent makes the child feel positive about him or herself, which influences the child in two ways: (a) it raises the esteem/belief state cbs + (purple) of the child. Secondly, the child realizes/conceptualizes through the prefrontal cortex state cPFC (green) and makes feel as ‘valuable’ at *t* = 11. So, under the parental influence, the child starts to mimic the parent around time point *t* = 15–50, feels rewarded (cstriatum: brown bold), and is happy about it (ces_happy_: mustard bold). However, until now the child is just reflecting his parents’ social behaviors, so the own feelings related to self-rewarding behavior are not activated yet. This mimicking behavior stays till *t* ≈ 280, so the values for the both of the states stay till 0.5.

After time point *t* = 280, the feelings of self-reward (fs_reward_: greyish blue) and self-love(fs_love_: maroon) start to increase, due to the activations in insula (cinsula: light blue). This behavior is expected, as the child is also learning (via **W**_4_) that mimicking the parent can be rewarding. At this point, the child starts to reflect the learned narcissism by his or her behaviors (social contagion). Therefore, activations in the self-rewarding states (fs_reward_, fs_love_, cinsula, along with cstriatum) cause the states cstriatum and ces_happy_ to get elevated (value > 0.95) at time point (*t* ≈ 300). It is to be noted that the belief state also reflects the learning behavior over time, and therefore it also goes to value = 1 at time point *t* = 290–300, showing that the child has become a narcissistic soul.

Here, the dotted lines show the dynamics of the connection weights through **W**_*i*_ states, where *i* = 2, 3, 4, 5, and 6. These dynamic states reflect the hebbian learning behavior, for example it can be seen that **W**_4_ (**W**_striatum,insula_) starts to grow at *t* > 50, as it is responsible for the child’s awareness [47]. This influences **W**_5_ and **W**_6_ (i.e., ${\mathbf{W}}_{{\rm fs}_{\rm reward},{\rm striatum} }$ and ${\mathbf{W}}_{{\rm fs}_{\rm love},{\rm striatum} }$) to learn over of time. Similarly, state **W**_2_ (${\mathbf{W}}_{{\rm bs},{\rm fs} _{\rm love}}$) and **W**_3_ (${\mathbf{W}}_{{\rm fs}_{\rm love},{\rm bs} }$) reflect the same behaviors around time *t* ≈ 280, which determine the adaptive connections (cbs  cfs_love_ and cfs_love_  cbs) at Level I.

To illustrate the control over the dynamics of **W**-states from Level III, it is nice to observe the role of the **M**- and **H**-states, which are shown by a dotted line and a line marked with stars, respectively. For example, by looking into W_4_, it can be observed that **M**_4_ and **H**_4_ reach high values (> 0.9) causing W_4_ to elevate at a faster speed than the rest of the W-states. As part of the circular causation, this influences the activation of the related postsynaptic state (i.e., insula), which in turn influences the other states (cfs_love_, cfs_reward_). These activations, make it possible for ‘neurons that fire together wire together’. Thus the activations in the respective **M**- and **H**-states enable to gain the connection strengths in: **W**_2_, **W**_3_, **W**_5_ and **W**_6_. As a combined effect, a sudden increase in **W**_3_, **W**_5_ and **W**_6_ can be observed at Level II between *t* = 250–300 (dotted lines). As a result, the respective connections get strong thus showing at Level I the social contagion behavior of a child (narcissistic influence).

#### Parental Influence ‘on’ the digital world

**Fig. 4:**
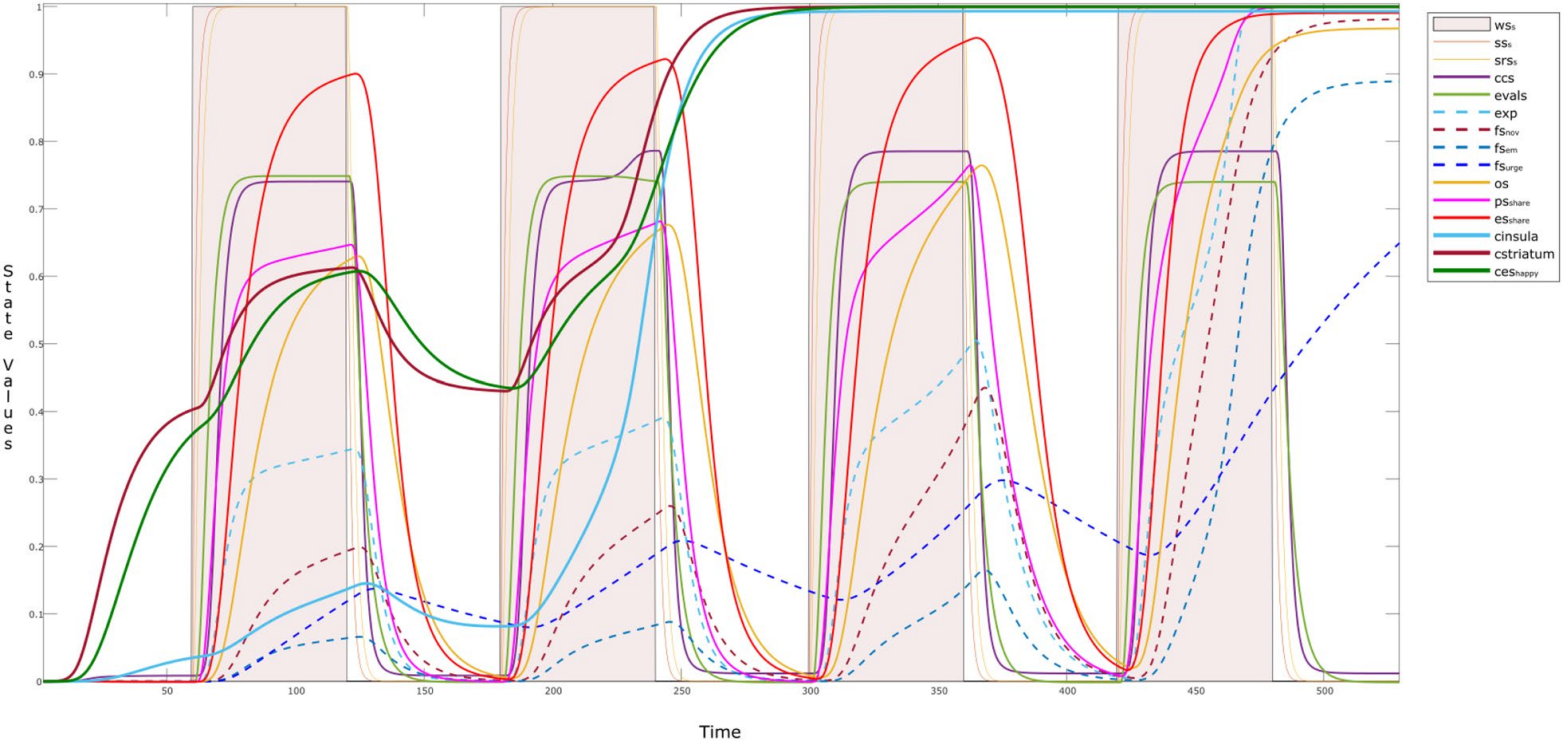
Episodes showing the behavior of the child while **a** not using social media, and **b** while using social media, with the tendency of the exhibition of parental narcissism

Here, we discuss the behavior of a child, who start using a social media apps (like Disney’s Club Penguin /Twitter/WhatsApp and so on), and is influenced by a happy narcissistic parent (es_happy_ = 1). To see how sharing over social media influences behavior of a child over time, we present the simulation in episodes. The two episodes include when a child (a) is not using social media, and (b) when (s)he is using social media. Each episode is differentiated by different colors such that, the episodes with white background show the behavior of a child while not using social media, for example, first episode is from *t* = 0–60. In contrast episodes with the colored background show the behavior when the child is using the social media, for example, from *t* = 60–120. It is to be noted that, length and duration of the episodes may vary and can be overlapping as well, however, for the sake of simplicity we kept them non-overlapping and with equal intervals (see Fig. 4).

The simulation starts with the episode when the child is ‘not using the social media’. The child already shows some influence of the parent before time point *t* = 60, through activations of self-rewarding states (bold curves—cstriatum: purple; ces_happy_: green; cinsula: cyan). The new episode starts when the child starts using social media (i.e., ws_s_ = 1) at *t* = 60. An example can be ‘*looking at a post*’, this will activate the child’s sensor state ss_*s*_ (orange) and representation states srs_*s*_ (yellow) around *t* = 61–65. After looking at the contents, the child evaluates it as an ‘*attraction seeking content*’ of the post, this activates his or her evaluation state eval_s_ (green), along with the control state ccs (purple), which is responsible to conceptualize the content. After evaluation, the child shares the content if it fulfills his or her criteria, which can be novelty, associated emotion and urge. By this we mean, if the child has a feeling that the content is novel (fs_nov_: brown dotted), and have some emotional association (fs_em_: red), the child’s urge (fs_urge_: dark blue dotted) to share the content gets higher. This criterion is learned by prior experiences and memories (exp: light blue dotted). An example of experience can be.

“which kind of posts gained maximum attention.”

An increase in the reward-related states is clearly observed here as well. Rather it would not be wrong to say that during the second episode: *t* = 120–180, the self-rewarding states seem lesser suppressed than the previous episode (*t* = 60–120). Clearly, this reflects the presence of attention-seeking behavior of the child or in other words, he is sharing the content for his narcissistic pleasure. Similar behavior is observed, till the self-rewarding states reach to their maximum value (value ≈1). It is interesting to observe that, unlike all other states, urge fs_urge_ to share the content does not drop to 0, while the child is not using social media. This make sense as the child enjoyed sharing the content, so (s)he will have an ongoing urge to use social media, and to get attention from his or her peers.

**Fig. 5:**
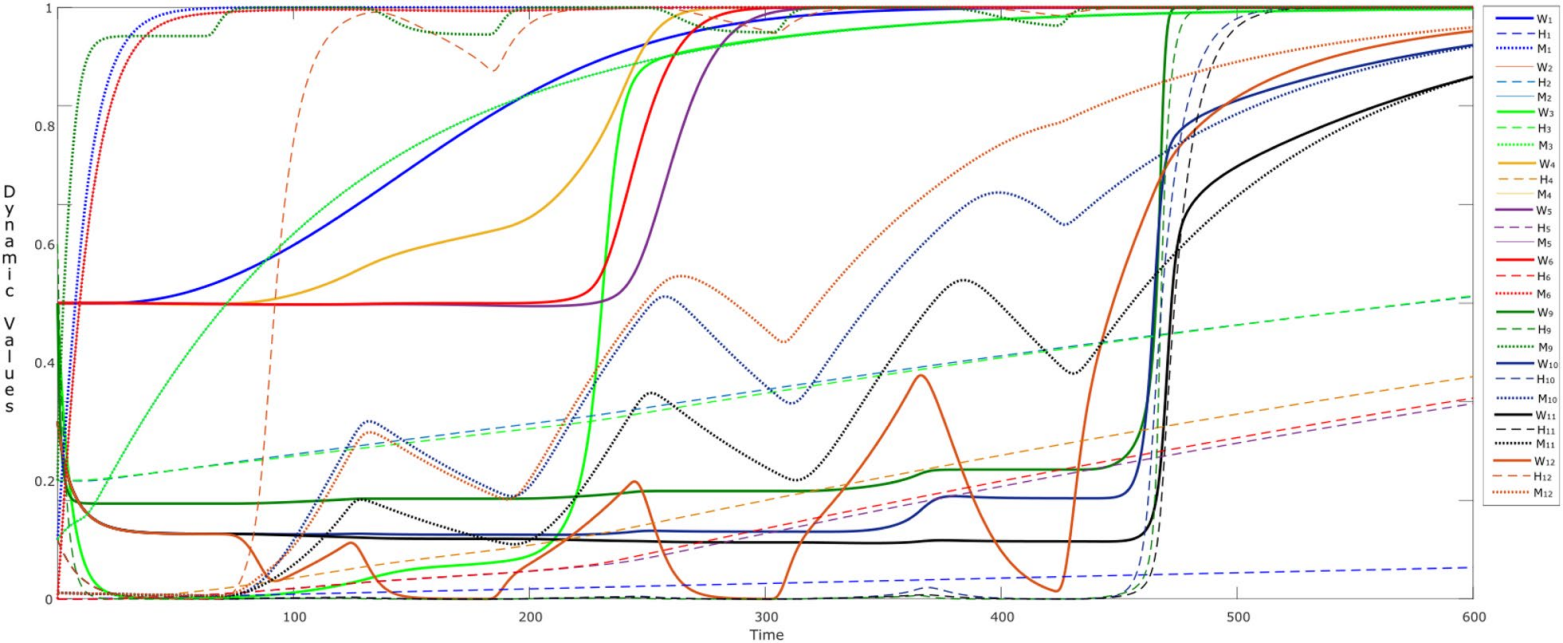
W-states during the alternative episodes addressed in Fig. 4

Interestingly, the effect of hebbian learning can naturally be seen in the alternative episodes, through different intensities or activation levels of the involved states. However, Fig. 5 provides more insights on the dynamic connection weights, which are changing over time. Here, the **W**-states related to self-rewarding behavior (W_*i*_: *i* = 2–6) continue to learn over time. It can be observed that there is a sudden increase in **W**_4_ (i.e., **W**_striatum,insula_) from *t* > 100 indicating that the child is getting aware of the self-rewarding behavior. This learning plays a vital role in activation and learning of the corresponding feelings (fs_reward_,fs_love_), and their connections through **W**_5_ (${\mathbf{W}}_{{\rm fs}_{\rm reward},{\rm striatum} }$) and **W**_6_ (${\mathbf{W}}_{{\rm fs}_{\rm love},{\rm striatum} }$) and so on.

While looking at the W-states related to the episode of ‘sharing content’ (i.e., **W**_*i*_: *i* = 9–12), we can see a gradual increase of almost all of the states from *t* = 0–450 till they reach to their maximum. However, this is not true for the **W**_12_ (${\mathbf{W}}_{{\rm urge},{\rm ps} _{\rm share}}$) as it drops to 0 in each episode of ‘not using the social media’, for example during *t* = 120–180; 240–300; and so on. The reason is that the urge of sharing content is almost new at the start of each episode (/exposure to social media) until he is a regular user of social media. By adjusting the **H**- (dotted lines) and **M**-states (small dots) for the **W**-states, learning can be controlled so that it accelerates or decelerates. The variations in reification states **M** for persistence and **H** for learning speed factor have their controlling effect on the dynamics of the respective **W**-states. It can be seen that, **H**_*i*_ and **M**_*i*_ (*i* = 9–12), show elevation with time. For example, with a gradual increase in **W**_11_ (${\mathbf{W}}_{{\rm fs}_{\rm em},{\rm ps}_{\rm share}}$:black) after *t* > 60, a periodic pattern is observed **M**_11_, indicating that in every episode of ‘a child using the social media’, the child is learning to establish an emotion related to some content (persisting the past experiences). However, **W**_11_ is able to reach its maximum value due to hebbian learning once **H**_11_ reaches its maximum value. The faster (learning) speed for **W**_11_ is also reflected in fs_em_  ps_share_.

### Exhibition of non-narcissistic behaviors

Here, we present how a child though nurtured by a narcissistic parent, blooms to his or her full potential. Therefore, in this section, we address two scenarios: a) how a child gains maturity with time, and b) how the digital world influences the child.

#### Maturity and the parental influence ‘off’ the digital world

According to Samuel Ullman

“maturity is the ability to think, speak, and act your feelings.”

In this section, we present the simulation where a child notices that his parent is a narcissist but resists to act like one. An example can be

“in a social gathering, unlike his parent, he prefers to remain unnoticed.”

**Fig. 6:**
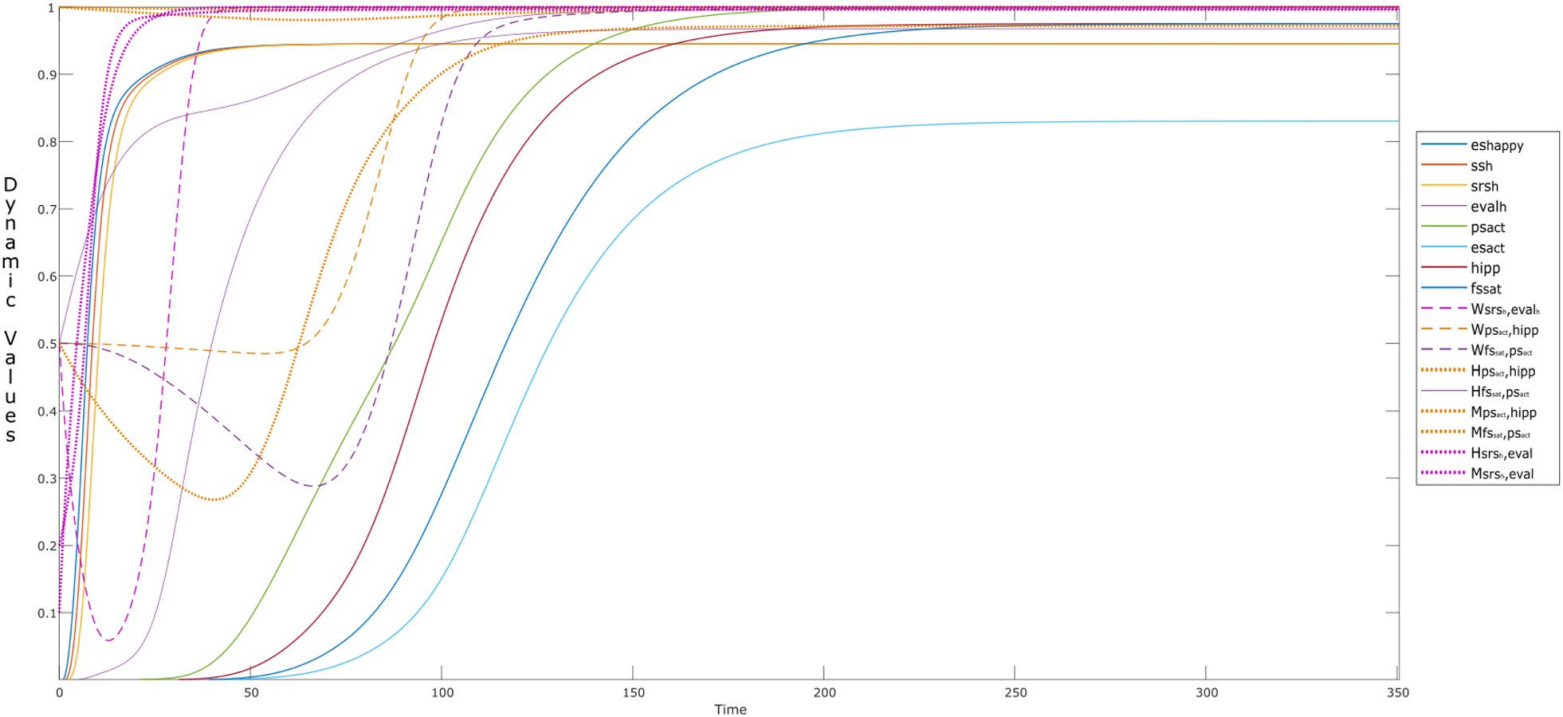
Dynamics observed when the child learns to be non-narcissistic given (s)he senses happiness (input: eh_happy_)

This scenario is addressed in Fig. 6, when a child starts getting the stimulus from a happy parent (es_happy_: blue), around time *t* < 25. The child senses (ss_h_: brown; srs_h_:mustard), and starts to learn about the parent’s behavior by the evaluation state eval_h_ (purple). This learning enables the child to act (cps_act_: green; es_act_: light blue) in such a way, which can please his or her own soul (fs_sat_: deep blue), rather than mirroring the parent, i.e., in a non-narcissistic way. An example can be.

“sitting on a couch, where no one notices him/her.”

The feeling of self-satisfaction comes with the past experiences and memories (hipp_1_: maroon), which he had over time.

Here, the learning of the connection weights (**W**-states) is shown by the dotted bold curves. It can be seen that **W**_1_ (${\mathbf {W}}_{{\rm srs}_{\rm h},{\rm eval} _{\rm h}}$:magenta) starts to learn around t ≈ 25, causing **W**_7_ (${\mathbf{W}}_{{\rm ps}_{\rm sat},{\rm hipp} }$:mustard), and **W**_8_ (${\mathbf {W}}_{{\rm fs}_{\rm sat},{\rm ps} _{\rm act}}$: purple) to learn as well by the time point *t* = 100. This learning behavior also can be observed through the activation levels of the action related states, i.e., ps_act_ (green) and es_act_ (blue). Here, it would be interesting to observe the ‘unlearning behavior’ of **W**_1_ (${\mathbf{W}}_{{\rm srs}_{\rm h},{\rm eval} _{\rm h}}$) and **W**_8_ (${\mathbf{W}}_{{\rm fs}_{\rm sat},{\rm ps} _{\rm act}}$). By this we mean the dip in the two curves, which shows that the child unlearns the behaviors related to self-satisfaction and evaluation from the past experiences and memories. Please note, **W**_7_ (${\mathbf{W}}_{{\rm ps}_{\rm sat},{\rm hipp} }$) does not show the same dip, which explains the learning of experiences in the child’s life over time. This is also observed through **M**- and **H**-states (small dotted curve) which play their role in controlling the elevation of the **W**-states. One thing to be noted here is that **M**_*i*_ (*i* = 7, 8) are initially set to 1. However, it would be interesting to note that, as the child is not satisfied initially, the values go down, and then the child learns the behavior which can satisfy him or her. The possible reason can be that the child was satisfied before (s)he started to evaluate the parent’s behavior. As the evaluation process starts and the child is going towards the action that can satisfy his/or her own soul, these states are increasing effecting the respective **W**-states (hebbian learning).

#### Non-narcissistic behaviors ‘on’ the digital world

In this section, we will see how the usage of social media does not elevate narcissism in a child under influence of a parent. To understand the learning behaviors, we again present episodes where: (a) a child is not on social media, and (b) a child is using social media.

**Fig. 7:**
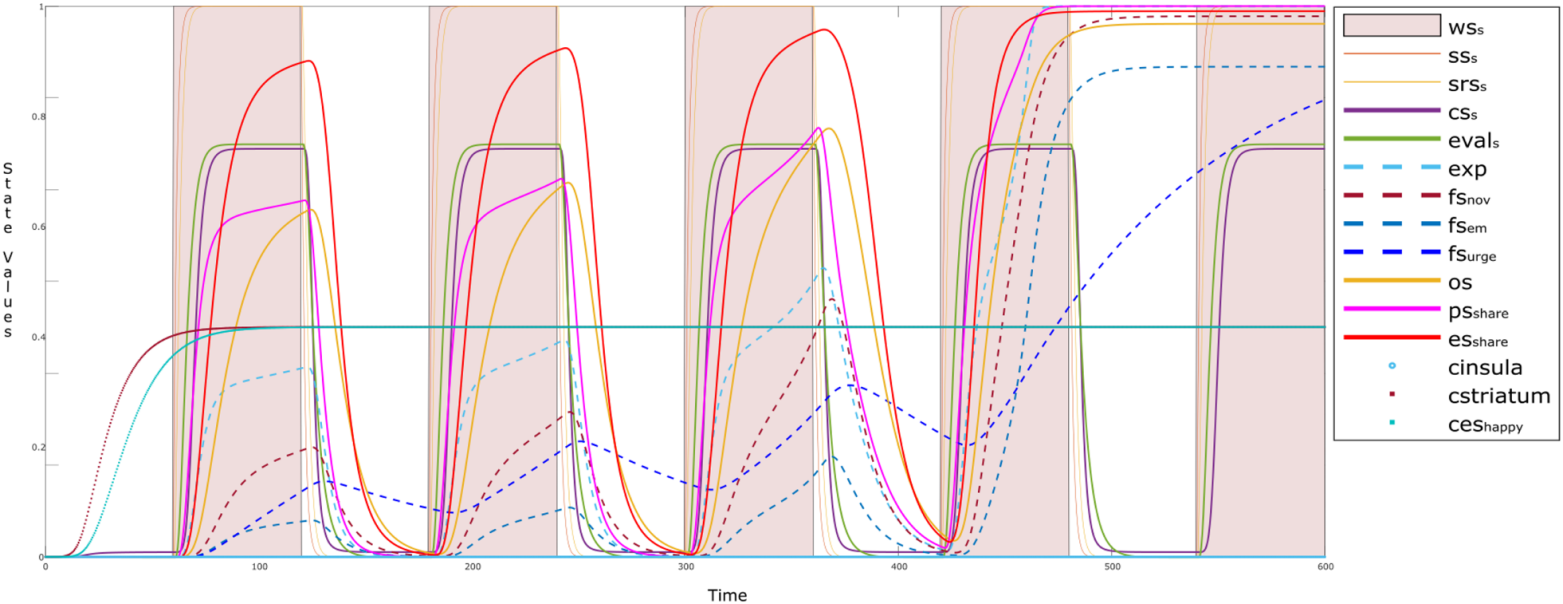
W-states during the alternative episodes addressed in Fig. 8

**Fig. 8:**
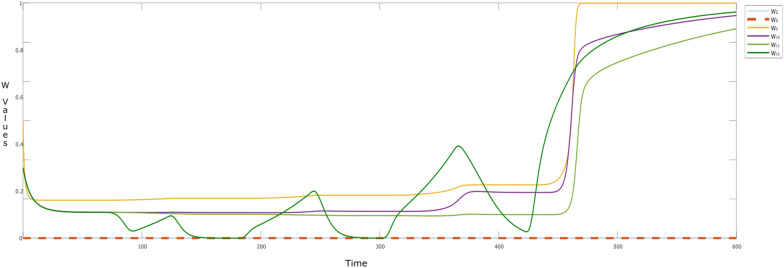
Episodes showing the behavior of the child while a) not using the social media, and b) while using the social media, without the tendency of narcissism

Most of the curves tend to show the same behavior like in Fig. 4, except for the self-rewarding behavior. Here, the activation levels of the related states do not vary over time (striatum; cinsula; ces_happy_; value = 0.42). Moreover, the learning behaviors of the **W**-states can be seen in Figs. 7 and 8 as well, where no dynamics are observed for **W**_2_ (${\mathbf{W}}_{{\rm bs},{\rm fs}_{\rm love} }$) and **W**_3_ (${\mathbf{W}}_{{\rm fs}_{\rm love},{\rm bs} }$) showing there is no activation for self-love (Fig. 8). This indicates that the child likes to share the content and feels happy like every child, but has less tendency of turning into an attention seeker or a narcissist as there is no gain in self-love. For Level III activation, they are similar to Fig. 5, however, as there is no learning for self-rewarding behavior, the related **M**- and **H**-states remain constant along with the **W**-states.

### Coping with a narcissistic parent

The simulations presented in Sect. 4.1 and 4.2 showed the behavior of a child while sensing the happiness of a narcissistic parent. What if the child is dealing with an unhappy parent or is facing an unpleasant situation? In this section, we will discuss what impression an unhappy parent can leave on the child’s brain. Also, we will address how the child can learn to cope with such a situation. Therefore, this section is divided into two subsections: (a) how an unhappy narcissistic parent influences the child, and (b) what can the child do to survive.

#### Unhappy face of a narcissistic parent

Literature shows that living with narcissistic abuse can be hazardous for mental and physical health [32, 34]. Consider a scenario which explains such a parent–child relationship:

“I was taken to a hospital when I was complaining about stomach and articular pain. I felt being under everyone’s feet and guilty of existing. I thought everyone would be better if I did not exist. I thought about suicide first time when I was nine. I just did not know how to do it.” [9]

Or, another example scenario can be:

“You dare not to express your feelings and you do not have sufficient connection with your emotions or yourself when a child or even as an adult. You learn how to hide your creativity and strength because they are being nullified, even envied at home.” [9]

**Fig. 9:**
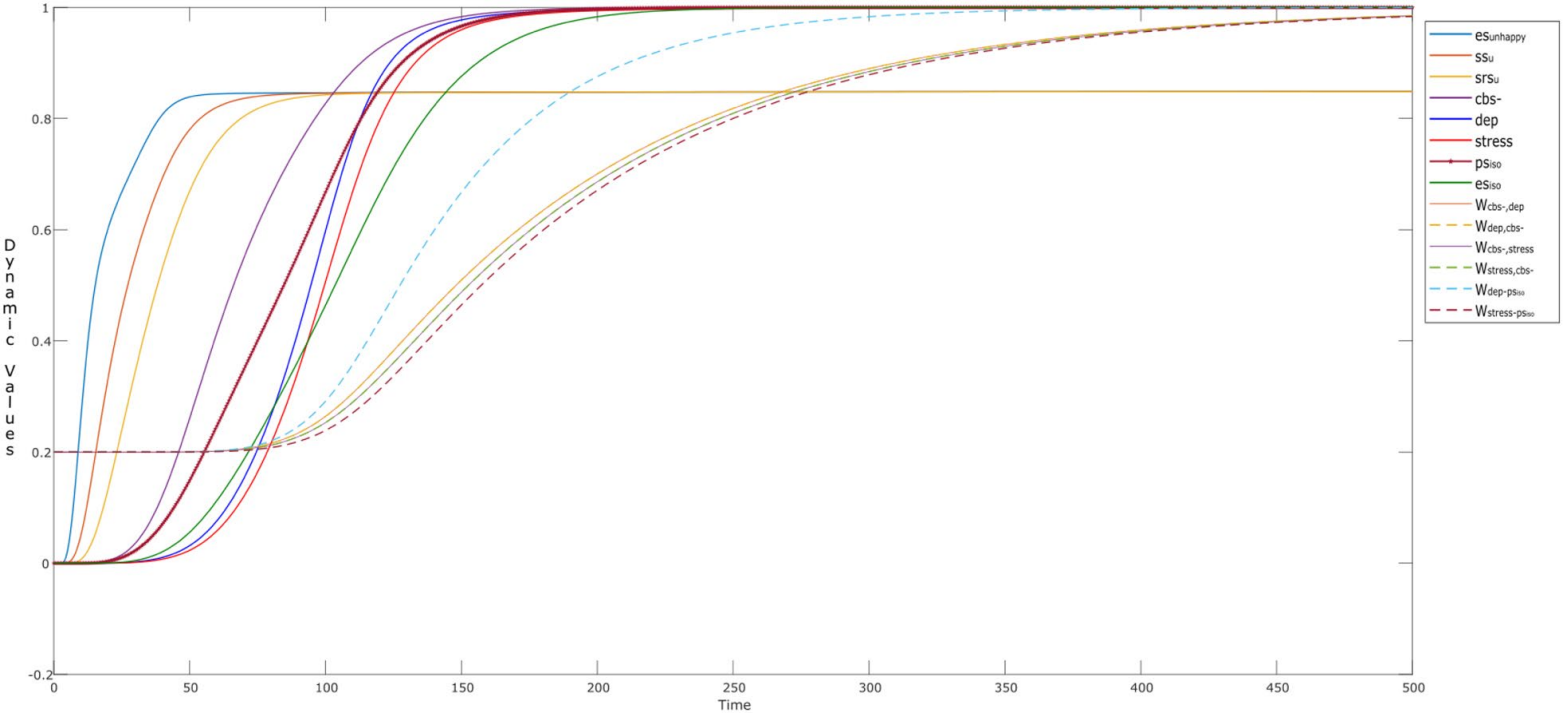
Behavior of an isolated child after the narcissistic abuse (input: es_unhappy_)

In order to explain such a scenario through simulation experiments, consider Fig. 9. Here, an unpleasant behavior (es_unhappy_: blue) of a narcissistic parent acts as a stimulus at *t* < 10. The child senses the stimulus through sensor state ss_u_ (brown) and sensory representation srs_u_ (mustard) states at time point *t* < 25. As addressed in the example, such behaviors make the child think for being responsible for every bad thing which can happen in the parent’s life. This may result in low esteem shown by cbs- (purple). Narcissistic abuse produces stress and depression (dep: deep blue), and as a result the child gets isolated (ps_iso_: magenta bold; es_iso_: green) from the parent as well as from the society, making the stress and depression elevated.

Here, hebbian learning of **W**-states: **W**_*i*_ (where *i* = 13—18) is also shown by dotted lines. These states do not change till *t* ≈ 80. However, when the child starts to get isolated, the **W**_*i*_ (*i* = 13—18) goes high, so are the involved states, i.e., depression (dep) and stress.

#### Coping with the unhappy face

Much research is done on how to cope with narcissists and to lead a healthy life [32, 34]. In this section, we present a scenario in which a child is learning to cope with the unhappy behavior of a narcissistic parent. A parent can be displeased by any situation around, which is causing mental distress to the child as well. However, when a child recognizes the narcissistic face of the parent, (s)he can cope with this pain. An example of self-supporting/coping behavior can be:

“After finding the illness called narcissism, I have been able to be stronger in front of my mother.” [9]

Or, another example scenario can be:

“The actual change toward better life started to happen when I moved away from my home place, to over 900 km away to study.” [9]

**Fig. 10:**
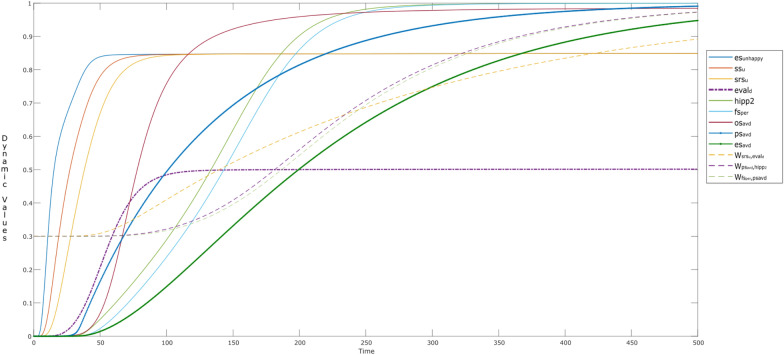
Coping with the narcissistic parent while (s)he is unhappy (input: es_unhappy_)

Figure 10 shows a simulation of the behavior of such a child. Whenever a parent shows displeasing behavior to the child (es_unhappy_: blue), the child senses it (ss_u_: brown; srs_u_: mustard). Gradually, the child evaluates and learns to recognize it as an intense displeasure displayed by the parent who is a narcissist, through the child’s evaluation state eval_d_ (purple dotted) around time point *t* = 25–70. The child realizes the parent’s nature, so starts to avoid the parent (ps_avd_: bold blue; and es_avd_: bold green). This is a conscious step which the child takes to survive, represented by the ownership state os_avd_ (maroon). Another example of avoidance behavior (avoidance strategy) can be something like:

“In order to survive, you have to shrink yourself when near a narcissist.” [9]

Or the child can choose to modify the situation (situation modification) to solve this problem like:

“I have chosen such work fields and places that have needed me working in shifts all the time. So that I could be away from home. If I tell her I am at home during the weekdays, she can call me 5 am asking for help. I went to work on sea so that I could not be reached at all. I worked at the ship for two years non-stop.” [9]

To survive, the child acts in a persuasive manner, so has to stay and feel firm (fs_per_: blue) in his or her actions. Also escaping from the parent makes him learn different workouts with different experiences and memories (hipp_2_: green), related to survival with the hot-headed parent. This is represented by the learning of connection weights, **W**_*i*_ (*i* = 19–21) from time point *t* = 45 for **W**_19_ (${\mathbf{W}}_{{\rm srs},{\rm eval}_{\rm d} }$: orange dotted), which gradually increases with time.

### Effects of coping behaviors

To break the cycle of continuous stress and anxiety, a child needs to learn for self-support and go for some problem-solving techniques by him or herself, or by the help of a trust-worthy person. Some examples of such techniques can be keeping a journal to identify behavioral changes for him or herself and for the parent. Another can be doing the deep breathing exercises or mindfulness, for example:

“Nature and nearby woods offered me a safe place to be. I used to play in the woods often alone. I thrived smelling the woods only because nothing in there intimidated or blamed me.” [9]

**Fig. 11:**
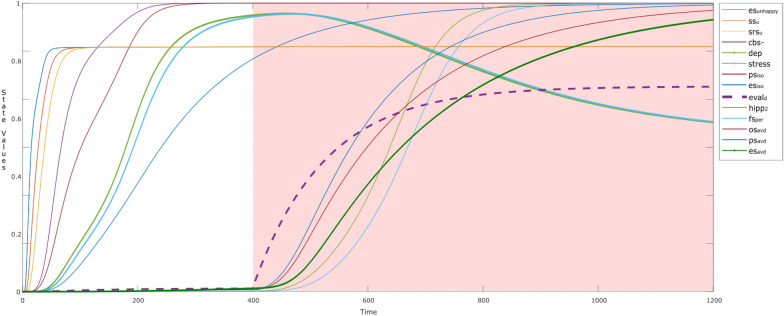
Coping behaviors reduce stress and depression/anxiety caused by narcissistic abuse

The effects of such problem-solving techniques can be observed by the simulation addressed in Fig. 11. Initially as addressed in Sect. 4.3.1 (white background), the child is feeling depression (dep: bold green) and stress (stress: bold blue). However, around time point *t* = 400 (colored background), the child learns to conceptualize the behavior of the parent (eval: purple dotted). As a result, the subsequent states ps_avd_ (blue), os_avd_ (maroon), hipp_2_ (green), and es_avd_ (bold green) are activated. At this point the opted strategy can be the choice of problem-solving technique (like breathing, mindfulness, and so on) through preparation and execution states ps_avd_ (maroon) and es_avd_ (blue) after *t* = 400. As an effect of activation of es_avd_, a gradual decrease can be observed in the levels of stress (bold blue) and depression/anxiety (dep:green).

**Fig. 12:**
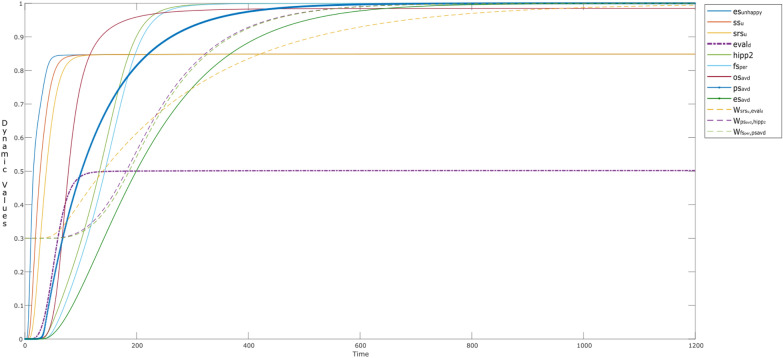
Child trying to cope with a firm behavior, showing no further dynamics

The firmness of the coping behavior can be reflected in Fig. 12, which shows the scenario addressed in Sect. 4.3.2 No further learning can be observed, due to past experiences and memories, so the states related to the child’s behavior gains equilibrium (therefore Eq. 1 becomes: *Y(t* + **Δ***t)* = *Y(t)*) without showing any further dynamics. This indicates that the child has learned the coping behaviors for his or her endurance.

## Discussion and conclusion

In this study, we presented an adaptive agent model of a child, who is influenced by a parent modeled as a second-order adaptive network model. It addresses adaptive cognitive and social processes, which are involved in preparing a child to act in a narcissistic or non-narcissistic way. We presented three types of behaviors through simulation experiments. Firstly, we presented how a child can learn to act in a narcissistic way. This is discussed under the parental influence while (a) being offline and; while (b) using the social media. It was observed, that in both of the scenarios nurturing behavior of a parent was reflected and the child tried to imitate the parent’s behavior both online and offline. Secondly, we showed how kids can behave in a non-narcissistic way under the parental influence. To address this, (a) we presented the child’s learning behavior in which (s)he chooses not to be like the parent and (b) change of behavior while using or not using social media. In these scenarios, it was observed, that the child did not go for narcissistic pleasure, but tried to opt for the choices in which (s)he was not imitating the parent. Lastly, we presented how an unpleasant behavior of a narcissistic parent can influence the child, and how different regulation strategies can help the child to recover from such a situation.

Our work has some limitations, we did not study many other factors that can influence a child along with the parental influence. For example, how the social relationship of a child with other family members or friends can influence him. Another example can be how the sensitivity/vulnerability of a child can influence him or her. Also, it would be interesting to see how child narcissism can influence a parent and how this loop will go on, or how personality development programs can influence the child. Moreover, this model needs to be validated through the empirical data according to the designed model. Although, some psychological studies are available with data related to child narcissism; however they do not mention any details with reference to the narcissism of the parents [6] or they are not changing over time [32].

Therefore, as the future work of our study, we aim to look into other psychological and social aspects of the behavior of a child, with a narcissistic parent. Moreover, we would also like to incorporate other factors like sensitivity or vulnerability of a child. Moreover, we would like to collect and study empirical data to validate our model. This validation process would reveal further behaviors of a child. In the end, by studying these behaviors, we aim to design and investigate the support strategies further, which can provide support to such a child, to become a better human.

## Data Availability

The data and materials used for analysis and development of results is available here.

## Appendix

### Numerical relevance of the model

The mathematical representation of a reified or self-modeling network architecture in terms of its network characteristics can be explained as follows [11]:

1. At every time point *t*, the activation level of state *Y* at time *t* is represented by *Y(t)*, with the values between [0,1].
2. The single impact of state *X* on state *Y* at time *t* is represented by **impact**_*X,Y*_*(t)* = ω_*X,Y*_* X(t)*; where *ω*_*X,Y*_ is the weight of connection *X* → *Y*. All single impacts for a given state *Y* are aggregated by a combination function ***c***_*Y*_**(..)**; see below.
3. Specific states are used to model specific types of network adaptation, where network characteristics such as connection weights or combination functions are dynamic. For example, **W**_*X,Y*_ represents an adaptive connection weight **ω**_*X,Y*_**(t)** for the connection *X* → *Y*, while **H**_*Y*_ represents an adaptive speed factor **η**_*Y*_*(t)* of state *Y.* Similarly, **C**_*i,Y*_ and **P**_*i,j,Y*_ represent adaptive combination functions ***c***_*Y*_**(**.., *t)* over time and their parameters, respectively. Combination functions are built as a weighted average from a number of basic combination functions bcf_*i*_(..) from a library, which take parameters *P*_*i,j,Y*_ and values *V*_*i*_ as arguments. For adaptive network models in which network characteristics are dynamic as well, the universal combination function ***c****_*Y*_**(..)** used for any state *Y* is defined as:
  $${\mathbf{c}}{^*}_{Y} (S,C_{1} ,...,C_{m} ,P_{1,1} ,P_{2,1} ,...,P_{1,m} ,P_{2,m} ,V_{1} ,...,V_{k} ,W_{1} ,...,W_{k} ,W) = W + S[C_{1} {\text{bcf}}_{1} (P_{{{1},{1}}} ,P_{{{2},{1}}} ,W_{{1}} V_{{1}} , \ldots ,W_{k} V_{k} ) + ... + C_{m} {\text{bcf}}_{m} (P_{{{1},m}} ,P_{{{2},m}} ,W_{{1}} V_{{1}} ,...,W_{k} V_{k} )]/(C_{{1}} + ... + C_{m} ){-}W],$$
  where at time *t*:
4. variable *S* is used for the speed factor reification **H**_*Y*_*(t).*
5. variable *C*_*i*_ for the combination function weight reification **C**_*i,Y*_*(t)*.
6. variable *P*_*i,j*_ for the combination function parameter reification **P**_*i*,*j*,*Y*_*(t)*.
7. variable *V*_*i*_ for the state value *X*_*i*_*(t)* of base state *X*_*i*_*.*
8. variable *W*_*i*_ for the connection weight reification **W**_*Xi,Y*_*(t).*
9. variable* W* for the state value *Y(t)* of base state *Y*.
  4. Based on the above universal combination function, the effect on any state *Y* after time Δ*t* is computed by the following *universal difference equation* as:
  $$Y(t + \Delta t) = Y(t) + [{\mathbf{c}}{^*}_{Y} ({\mathbf{H}}_{Y} (t),{\mathbf{C}}_{1,Y} (t),...,{\mathbf{C}}_{m,Y} (t),{\mathbf{P}}_{1,1} (t),{\mathbf{P}}_{2,1} (t),...,{\mathbf{P}}_{1,m} (t),{\mathbf{P}}_{2,m} (t),X_{1} (t),...,X_{k} (t),{\mathbf{W}}_{{X_{1} ,Y}} (t),...,{\mathbf{W}}_{{X_{k} ,Y}} (t),Y(t)) - Y(t)]\Delta t,$$

which also can be written as a universal differential equation:

$${\mathbf{d}}Y(t)/{\mathbf{d}}t = {\text{c}}{^*}_{Y} ({\mathbf{H}}_{Y} (t),{\mathbf{C}}_{1,Y} (t),...,{\mathbf{C}}_{m,Y} (t),{\mathbf{P}}_{1,1} (t),{\mathbf{P}}_{2,1} (t),...,{\mathbf{P}}_{1,m} (t),{\mathbf{P}}_{2,m} (t),X_{1} (t),...,X_{k} (t),{\mathbf{W}}_{{X_{1} Y}} (t),...,{\mathbf{W}}_{{X_{k,} Y}} (t),Y(t)) - Y(t).$$

## Abbreviations

ACC: Anterior Cingulate Cortex: Brain Part
GABA: γ-aminobutyric acid
CBT: Cognitive Behavioral Therapy
ws: World state
ss: Sensory state
srs: Sensory representation state
eval_h_: Evaluation state of happiness
eval_d_: Evaluation state of disorder
cbs: Child belief state
PFC: Prefrontal Cortex: Brain Part
os: Ownership state
ps: Preparation state
es: Execution state
act: Action
h: Happiness
u: Un-happiness
fs: Feeling state
hipp: Hippocampus: Brain Part
sat: Satisfaction
exp: Experience
nov: Novelty of content
em: Emotional association with the content
urge: Urge associated with the content
cs: Control state
dep: Depression related state
stress: Stress related state
avd: Avoiding a situation

## List of symbols

μ: Persistence
η_Y_: Speed factor of state Y
ω_X,Y_: Connection weight of a connection from X to Y
c_Y_: Combination function for state Y
P_i,j_: Combination function parameter reification
W_1_;${\rm W}_{{\rm srs}_{\rm h},{\rm eval} _{\rm h}}$: Reification state for the weight of the connection srsh→evalh
W_2_;${\rm W}_{{\rm fs}_{\rm love},{\rm bs} }$: Reification state for the weight of the connection fs_love_→cbs+
W_3_;${\rm W}_{{\rm bs},{\rm fs} _{\rm love}}$: Reification state for the weight of the connection cbs+→fs_love_
W_4_;W_striatum__,__insula_: Reification state for the weight of the connection striatum→insula
W_5_;${\rm W}_{{\rm fs}_{\rm reward},{\rm striatum} }$: Reification state for the weight of the connection fs_reward_→striatum
W_6_;${\rm W}_{{\rm fs}_{\rm love},{\rm striatum} }$: Reification state for the weight of the connection fs_love_→ striatum
W_7_;${\rm W}_{{\rm fs}_{\rm sat},{\rm hipp} }$: Reification state for the weight of the connection fs_sat_ → hipp
W_8_;${\rm W}_{{\rm fs}_{\rm sat},{\rm ps} _{\rm act}}$: Reification state for the weight of the connection fs_sat_→ ps_sat_
W_9_;${\rm W}_{{\rm ps}_{\rm share},{\rm exp} }$: Reification state for the weight of the connection ps_share_→exp
W_10_;${\rm W}_{{\rm fs}_{\rm nov},{\rm ps} _{\rm share}}$: Reification state for the weight of the connection fs_nov_→ ps_share_
W_11_;${\rm W}_{{\rm fs}_{\rm em},{\rm ps} _{\rm share}}$: Reification state for the weight of the connection fs_em_→ ps_share_
W_12_;${\rm W}_{{\rm urge,ps}_{\rm share}}$: Reification state for the weight of the connection urge→ ps_share_
W_13_;W_cbs__,__dep_: Reification state for the weight of the connection cbs→ dep
W_14_;W_dep__,__cbs_: Reification state for the weight of the connection dep→ cbs+
W_15_;W_cbs__,__stress_: Reification state for the weight of the connection cbs+→stress
W_16_;W_stress__,__cbs_: Reification state for the weight of the connection stress→ cbs+
W_17_;${\rm W}_{{\rm dep,ps}_{\rm iso}}$: Reification state for the weight of the connection de→ ps_iso_
W_18_;${\rm W}_{{\rm stress,ps}_{\rm iso}}$: Reification state for the weight of the connection stress→ ps_iso_
W_19_;${\rm W}_{{\rm srs}_{\rm u},{\rm eval} _{\rm d}}$: Reification state for the weight of the connection srs_u_→ eval_d_
W_20_;${\rm W}_{{\rm ps}_{\rm avd},{\rm hipp} _{\rm 2}}$: Reification state for the weight of the connection ps_avd_→ hipp_2_
W_21_;${\rm W}_{{\rm fs}_{\rm per},{\rm ps} _{\rm avd}}$: Reification state for the weight of the connection fs_per_→ ps_avd_
M: Reification state for the persistence
H: Reification state for the speed factor
love: Self-love
reward: Self-reward
